# Navigating Authorship Ethics in Research: Challenges and Responsibilities

**DOI:** 10.31729/jnma.8816

**Published:** 2024-11-30

**Authors:** Jay Bhushan Jha

**Affiliations:** 1 Associate Editor, Journal of Nepal Medical Association, Exhibition Road, Kathmandu, Nepal

While starting research of any kind, authors must be involved in the conceptualization or design or acquisition, analysis, or interpretation of data. They should also draft or review the drafted work critically. A final approval for publication and accountability and taking responsibility of the work is a must for an author.^1^

Authorship of any article has academic, social, and financial implications, which implies the responsibility for their work. Contributions should be made by every author to ensure fairness, especially in low- and middle-income countries. Journals adhere and should be encouraged to follow International Committee of Medical Journal Editors (ICMJE) criteria to differentiate between authors and contributors, which provides accountability and proper recognition of authors.^1^

Authorships are challenging in relations like student-faculty or mentor-mentee relationships, where power imbalance is evident. To prevent the authorship status early and respectful negotiations are the key. Authorship reflects the contributions made by the author, as the corresponding author are responsible for answering queries of the journal and is the first responsible contact ensuring the integrity of the work. These practices should be applicable in projects where there is inclusion of multiple authors or projects.^2^

Various challenges are faced while publishing research. Authors/ Researchers are often lured towards the academic reward system to publish in journals with high impact factors and in journals that are indexed in multiple libraries. This may lead researchers to prioritize quantity rather than quality. Even some researchers choose to hide the conflict of interest, which can be financial, professional or personal, which somewhere further compromises the integrity of research. In the process of showing extravagant results reporting biases, novel findings are often presented by researchers, which affects the evidence that is present. Additionally, statistical and methodological issues, which include misuse of P-values, statistical tests and post-hoc tests, can contribute to misleading and overstating conclusions, which can affect the credibility of research.^3^

Another issue commonly seen in research articles is plagiarism. It is due to the misconception that copying the whole paragraph is acceptable if citation is done, which ultimately leads to rejection and retraction of articles. Plagiarism is attributed to various factors like poor time management, writing under stress and underdeveloped scientific writing. With a fear of perishing of an article, researchers try to publish their article quickly either to secure fund, or to prove their skill or to advance in their career, which often leads to intentional plagiarism.^4^ These practices can be avoided by properly citing the sources of information which ultimately increases transparency and credibility. Contribution of other should be given credit, citations and use of footnotes should be properly done according to journal guidelines. For elaborative discussion of other works, permission should be taken from the publisher or author. Self-plagiarism should also be avoided by proper paraphrasing and by seeking permission from publisher or copyright holder.^5^

**Types of Authorship Issues:**

1. **Gift Authorship:** If an individual is given the credit of author despite contributing to the work, it is termed as gift authorship. Various reasons like a reward to collaborator, return of favor and some other personal benefit can be reasons behind it.^6^
2. **Ghost Authorship:** In some instances, even if the individual has contributed to the research, data analysis, or writing of a manuscript, they may not be credited as an author or in the acknowledgment section. These commonly include junior researchers, resident doctors, and postdoctoral fellows, who are commonly excluded from authorship. Another form include, individual who were not part of the starting research group but helped in writing or editing the manuscript to improve the quality , which ultimatlely saved the researcher's time.^7^

## AUTHORSHIP GUIDELINES:

Various standard policies guide in maintaining the integrity and ethical standards of research publishing. Authors are expected to adhere to guidelines set by reputed institutions like ICMJE and the Committee of Publication Ethics (COPE), which provides standards for authorship, data transparency, conflict of interest disclosure, and ethical research practices. These guidelines help in accurate representation of work and contributions made by researchers and also help to define the conflict of interests.^1,2^

Journals also provide specific guidelines and submission requirements that should be followed by the author. These help authors refine their articles by formatting, following ethical considerations, and rules regarding plagiarism. Some journals also ask for CrediT statements to clarify every author's contribution to research.^8^

By adhering to both institutional and journal's guidelines, author can increase the credibility of their article and promote fairness in medical publishing.

**Integrity in Research:** For a researcher, integrity is about being honest and responsible of their action and work. This commitment is reflected in actions like writing research proposals, reporting data, representing contributions, fairness in Peer review, transparency in conflict of interest, adhering to ethical standards while dealing with human subjects and respecting mutual responsibilities.^9^

Journals and institutions aiming to create a transparent and ethical environment must continuously monitor structures, processes, policies, and procedures. A system to support leadership, respect for researchers, productive student-faculty or trainee-mentor relationship, adherence to research rules, and transparent conflict of interest should be created via investigations of misconduct, trainings on research integrity and evaluation of quality improvement.^9^

## TIPS FOR AUTHORS/RESEARCHERS:

**Clear Communication:** Clear communication between co-authors is important to understand roles and contributions. Regular meetings, detailed emails, and the formation of tools that track the progress can help achieve communication. This can prevent misunderstanding, can create accountability and foster a collaborative environment where contributions will be recognized and valued.

**Documentation:** Records of contributions and decisions related to authorship are crucial for transparency and accountability. This can be maintained by creating subheadings of the work of research and documenting the coauthor's contribution in each article.

**Training and Education:** Training programs on research ethics and authorship for new researchers can help them understand and ask valuable questions about ethical standards. These may help in adhering to rules of authorship, managing conflict of interest and being transparent.^10^

## References

[1] International Committee of Medical Journal Editors. Defining the Role of Authors and Contributors.. International Committee of Medical Journal Editors.

[2] COPE Council. (2019). COPE Discussion Document: Authorship.. Committee on Publication Ethics.

[3] Bradley SH, DeVito NJ, Lloyd KE, Richards GC, Rombey T, Wayant C (2020). Reducing Bias and Improving Transparency in Medical Research: A Critical Overview of the Problems, Progress and Suggested Next Steps.. J R Soc Med..

[4] Mohammed R, Shaaban O, Mahran D, Attellawy H, Makhlof A, Albasri A (2015). Plagiarism in Medical Scientific Research.. J Taibah Univ Med Sci..

[5] Dhammi IK, Ul Haq R (2016). What is Plagiarism and How to Avoid it?. Indian J Orthop..

[6] Charlesworth Author Services. (2019). Ethics in Academic Publishing: Understanding 'Gift' Authorships.. Charlesworth Author Services..

[7] Bavdekar SB (2012). Authorship Issues.. Lung India..

[8] Elsevier. Author Policies and Guidelines: CRediT Author Statement.. Elsevier.

[9] National Research Council (US) and Institute of Medicine (US) Committee on Assessing Integrity in Research Environments. (2002). Integrity in Scientific Research: Creating an Environment That Promotes Responsible Conduct.. National Academies Press (US).

[10] Knight J (2023). Evaluating the Impacts of a Research Ethics Training Course on University Researchers.. Soc Sci..

